# Young-Onset Dementia Among Individuals With History of Preeclampsia

**DOI:** 10.1001/jamanetworkopen.2024.12870

**Published:** 2024-05-30

**Authors:** Valérie Olié, Grégory Lailler, Marion J. Torres, Nolwenn Regnault, Laure Carcaillon-Bentata, Jacques Blacher

**Affiliations:** 1 Santé Publique France, Saint-Maurice, France; 2 Université Paris Est, Créteil, France; 3 Pharmacoepidemiologie, Bordeaux, France; 4 Centre de Diagnostic et de Thérapeutique, Hôtel Dieu, Assistance Publique–Hôpitaux de Paris, Université Paris Cité, Paris, France

## Abstract

This cohort study assesses the risk of developing early-onset dementia in individuals who had preeclampsia and other hypertensive disorders during pregnancy.

## Introduction

Preeclampsia remains a major cause of maternal and fetal morbidity and mortality, affecting 2% to 5% of pregnancies.^1^ Many studies have shown this disorder has consequences that last decades after pregnancy, with an increased risk of cardiovascular and kidney morbidity and mortality in individuals with a history of preeclampsia.^2,3^ Recently, those with preeclampsia were described as having a 3-fold higher risk of late-onset vascular dementia after a mean follow-up of 20 years.^4^ This cohort study investigated whether preeclampsia and other hypertensive disorders of pregnancy (HDPs) were associated with an increased risk of young-onset dementia years after the pregnancy.

## Methods

Data were obtained from the Conception study, a nationwide prospective cohort study using data from the French National Health Insurance Information System. This database included all deliveries in France after 22 weeks’ gestation between January 1, 2010, and December 31, 2018. The French National Public Health Agency has permanent access to the pseudonymized health care reimbursement data in application of the provisions of the French Public Health Code and authorization to perform studies from the French Data Protection Authority, with rules and criteria similar to the Declaration of Helsinki. The French Data Protection Authority approved this study and waived the informed consent requirement because it used anonymized data.

All individuals aged 30 years or older without a history of dementia were identified and followed-up from delivery to December 31, 2021. Hazard ratios (HRs) were calculated using crude and adjusted Cox proportional hazards regression models. In all models, exposures to HDPs were treated as time-varying variables and maternal age as a time scale. Models were adjusted for obesity, diabetes, tobacco smoking, drug or alcohol addiction, and social deprivation. Data analysis was performed between March and July 2023 using SAS Enterprise Guide 7.1 (SAS).

## Results

Of the 1 966 323 individuals (mean [SD] maternal age, 34.6 [3.3] years) included, 128 (<1%) developed dementia. Dementia was identified using primary diagnosis for hospitalization during a mean (SD) follow-up of 9.0 (2.6) years. Patients who developed young-onset dementia were older (36.4 years vs 34.6 years), were more frequently socially deprived (13.3% vs 12.0%), were smokers (14.8% vs 9.0%), and had diabetes (2.3% vs 0.7%) compared with those without dementia (Table 1). Preeclampsia was associated with an increased risk of young-onset dementia compared with pregnancy without HDP (HR, 2.65; 95% CI, 1.34-5.25) (Table 2). Risk of young-onset dementia was even higher when preeclampsia occurred before 34 weeks’ gestation (HR, 4.15; 95% CI, 1.30-13.14) or was superimposed on chronic hypertension (HR, 4.76; 95% CI, 1.49-15.22). In contrast, severe preeclampsia was not associated with young-onset dementia.

**Table 1.** Baseline Characteristics of Patients Included

Characteristics^a^	No. (%)	
	Without dementia (n = 1 966 195)	With dementia (n = 128)
Mean (SD) maternal age, y	34.6 (3.3)	36.4 (4.1)
Median (IQR) maternal age, y	34.0 (32.0-37.0)	36.0 (33.0-39.0)
Mean (SD) follow-up, y	9.0 (2.6)	10.0 (2.2)
Median (IQR) follow-up, y	9.41 (7.0-11.2)	10.7 (8.8-11.7)
Social deprivation	236 814 (12.0)	17 (13.3)
Obesity	86 632 (4.4)	4 (3.1)
Diabetes	13 039 (0.7)	3 (2.3)
Tobacco smoking or addiction	176 034 (9.0)	19 (14.8)
Hypertensive disorders of pregnancy		
Chronic hypertension before pregnancy	48 745 (2.5)	7 (5.5)
Gestational hypertension	115 545 (5.9)	11 (8.6)
Preeclampsia	56 890 (2.9)	9 (7.0)

^a^ Data on social deprivation, obesity, diabetes, and tobacco smoking or addiction were collected in the year before or during pregnancy.

**Table 2.** Crude and Adjusted Hazard Ratio of Young-Onset Dementia by Hypertensive Disorders of Pregnancy

Covariate	No. of dementia cases	Crude HR (95% CI)^a^	Adjusted HR (95% CI)^b^
Without hypertensive disorders of pregnancy	108	1 [Reference]	1 [Reference]
Gestational hypertension	11	1.46 (0.79-2.72)	1.46 (0.78-2.71)
Preeclampsia	9	2.65 (1.34-5.25)	2.65 (1.34-5.22)
Severe	2	1.44 (0.36-5.82)	1.45 (0.36-5.88)
Early	3	4.16 (1.32-13.10)	4.15 (1.30-13.14)
Superimposed on chronic hypertension	3	4.84 (1.54-15.24)	4.76 (1.49-15.22)

Abbreviation: HR, hazard ratio.

^a^ Hazard ratios were calculated using crude and adjusted Cox proportional hazards regression models. In all models, the exposures to hypertensive disorders of pregnancy were treated as time-varying variables and maternal age as time scale.

^b^ Adjusted on obesity, diabetes, tobacco smoking, drug or alcohol addiction, and social deprivation.

## Discussion

Although earlier studies have already reported cognitive function impairment and white matter lesions associated with preeclampsia, the present study is the first to show an increase in early-onset dementia risk.^5^ The results suggested a dose-dependent association, with higher HRs for early-onset preeclampsia and superimposed preeclampsia. The nonsignificant result regarding severe preeclampsia could be associated with the low statistical power in this subgroup, adding to the currently limited knowledge about risk factors of young-onset dementia.^6^ A study limitation was that the identification of dementia by hospital diagnosis may have substantially underestimated the incidence of this disease.

More studies are needed to confirm these initial findings and to ascertain whether preeclampsia is an independent risk factor for dementia and whether preeclampsia and young-onset dementia share common risk factors or a physiological susceptibility to dementia unmasked by pregnancy. The results add early-onset preeclampsia to the list of lifelong disease risks or health care implications of having had preeclampsia.

## Notes

Supplement.
Data Sharing Statement

## References

[1] Chappell LC, Cluver CA, Kingdom J, Tong S. Pre-eclampsia. Lancet. 2021;398(10297):341-354. doi:10.1016/S0140-6736(20)32335-7 34051884

[2] Schliep KC, Mclean H, Yan B, . Association between hypertensive disorders of pregnancy and dementia: a systematic review and meta-analysis. Hypertension. 2023;80(2):257-267. doi:10.1161/HYPERTENSIONAHA.122.19399 36345823 PMC9851987

[3] Boucheron P, Lailler G, Moutengou E, . Hypertensive disorders of pregnancy and onset of chronic hypertension in France: the nationwide CONCEPTION study. Eur Heart J. 2022;43(35):3352-3361. doi:10.1093/eurheartj/ehab686 34643681

[4] Schliep KC, Shaaban CE, Meeks H, . Hypertensive disorders of pregnancy and subsequent risk of Alzheimer’s disease and other dementias. Alzheimers Dement (Amst). 2023;15(2):e12443. doi:10.1002/dad2.12443 37223334 PMC10201212

[5] Beckett AG, McFadden MD, Warrington JP. Preeclampsia history and postpartum risk of cerebrovascular disease and cognitive impairment: potential mechanisms. Front Physiol. 2023;14:1141002. doi:10.3389/fphys.2023.1141002 37064920 PMC10102351

[6] Carcaillon-Bentata L, Quintin C, Boussac-Zarebska M, Elbaz A. Prevalence and incidence of young onset dementia and associations with comorbidities: a study of data from the French National Health Data System. PLoS Med. 2021;18(9):e1003801. doi:10.1371/journal.pmed.1003801 34555025 PMC8496799

