# Supplementary material for: Young-Onset Dementia Among Individuals With History of Preeclampsia
Source: JAMA Netw Open. 2024 May 30;7(5):e2412870. doi: 10.1001/jamanetworkopen.2024.12870 (PMC11140526; doi:10.1001/jamanetworkopen.2024.12870)
Supplement: Supplement. — Data Sharing Statement [file jamanetwopen-e2412870-s001.pdf]

Data Sharing Statement

Olié. Young-Onset Dementia Among Individuals With History of Preeclampsia. *JAMA Netw Open*. Published May 30, 2024. doi:10.1001/jamanetworkopen.2024.12870

Data
Data available: No

Additional Information
Explanation for why data not available: The authors cannot share National Health Data System data as they are only available on a secure portal. Authorization to access this portal needs registration and clearance.
